# Validation of Serum Neurofilament Light Chain as a Biomarker of Parkinson’s Disease Progression

**DOI:** 10.1002/mds.28206

**Published:** 2020-08-15

**Authors:** Brit Mollenhauer, Mohammed Dakna, Niels Kruse, Douglas Galasko, Tatiana Foroud, Henrik Zetterberg, Sebastian Schade, Roland G. Gera, Wenting Wang, Feng Gao, Mark Frasier, Lana M. Chahine, Christopher S. Coffey, Andrew B. Singleton, Tanya Simuni, Daniel Weintraub, John Seibyl, Arthur W. Toga, Caroline M. Tanner, Karl Kieburtz, Kenneth Marek, Andrew Siderowf, Jesse M. Cedarbaum, Samantha J. Hutten, Claudia Trenkwalder, Danielle Graham

**Affiliations:** 1 Department of Neurology, University Medical Center Goettingen, Goettingen, Germany; 2 Paracelsus-Elena Klinik, Kassel, Germany; 3 Department of Neuropathology, University Medical Center Goettingen, Goettingen, Germany; 4 Department of Neurosciences, University of California, San Diego, San Diego, California, USA; 5 Department of Medical and Molecular Genetics, Indiana University School of Medicine, Indianapolis, Indiana, USA; 6 Clinical Neurochemistry Laboratory, Sahlgrenska University Hospital, Gothenburg, Sweden; 7 Department of Psychiatry and Neurochemistry, Sahlgrenska Academy at the University of Gothenburg, Mölndal, Sweden; 8 Department of Neurodegenerative Disease, UCL Institute of Neurology, London, United Kingdom; 9 UK Dementia Research Institute at UCL, London, United Kingdom; 10 Department of Medical Statistics, University Medical Center Goettingen, Goettingen, Germany; 11 Biostatistics, Biogen, Cambridge Massachusetts, USA; 12 The Michael J. Fox Foundation for Parkinson’s Research, New York, New York, USA; 13 Department of Neurology, University of Pittsburgh, Pittsburgh, Pennsylvania, USA; 14 Department of Biostatistics, College of Public Health, University of Iowa, Iowa City, Iowa, USA; 15 Molecular Genetics Section, Laboratory of Neurogenetics, National Institute on Aging, National Institutes of Health, Bethesda, Maryland, USA; 16 Parkinson’s Disease and Movement Disorders Center, Northwestern University Feinberg School of Medicine, Chicago, Illinois, USA; 17 Department of Neurology, Perelman School of Medicine, University of Pennsylvania, Philadelphia, Pennsylvania, USA; 18 Institute for Neurodegenerative Disorders, New Haven, Connecticut, USA; 19 Laboratory of Neuro Imaging, University of Southern California, Stevens Neuroimaging and Informatics Institute, Keck School of Medicine of University of Southern California, Los Angeles, California, USA; 20 Department of Neurology, University of California San Francisco, San Francisco, California, USA, and Parkinson’s Disease Research Education and Clinical Center, San Francisco Veterans Affairs Health Care System, San Francisco, California, USA; 21 Clinical Trials Coordination Center, University of Rochester Medical Center, Rochester, New York, USA; 22 Coeruleus Clinical Sciences LLC, Woodbridge, Connecticut, USA; 23 Discovery and Early Development Biomarkers, Biogen, Cambridge, Massachusetts, USA

**Keywords:** Parkinson’s disease/parkinsonism, cohort studies, outcome research

## Abstract

**Background:**

The objective of this study was to assess neurofilament light chain as a Parkinson’s disease biomarker.

**Methods:**

We quantified neurofilament light chain in 2 independent cohorts: (1) longitudinal cerebrospinal fluid samples from the longitudinal de novo Parkinson’s disease cohort and (2) a large longitudinal cohort with serum samples from Parkinson’s disease, other cognate/neurodegenerative disorders, healthy controls, prodromal conditions, and mutation carriers.

**Results:**

In the Parkinson’s Progression Marker Initiative cohort, mean baseline serum neurofilament light chain was higher in Parkinson’s disease patients (13 ± 7.2 pg/mL) than in controls (12 ± 6.7 pg/mL), *P* = 0.0336. Serum neurofilament light chain increased longitudinally in Parkinson’s disease patients versus controls (*P* < 0.01). Motor scores were positively associated with neurofilament light chain, whereas some cognitive scores showed a negative association.

**Conclusions:**

Neurofilament light chain in serum samples is increased in Parkinson’s disease patients versus healthy controls, increases over time and with age, and correlates with clinical measures of Parkinson’s disease severity. Although the specificity of neurofilament light chain for Parkinson’s disease is low, it is the first blood-based biomarker candidate that could support disease stratification of Parkinson’s disease versus other cognate/neurodegenerative disorders, track clinical progression, and possibly assess responsiveness to neuroprotective treatments. However, use of neurofilament light chain as a biomarker of response to neuroprotective interventions remains to be assessed.

Two major obstacles hamper the success of translational Parkinson’s disease (PD) research: (1) currently, no fluid biomarker for PD correlates with disease progression; and (2) a definite diagnosis of PD can only be made by autopsy, and the rate of clinical misdiagnoses, especially early in the disease course, is reported to be high.^1,2^ Therefore, biomarkers that could be used for diagnosis and as progression markers in PD are needed.

Neurofilaments are highly phosphorylated neuronal cytoskeleton components maintaining neuronal structure and determining axonal caliber. The 68-kDa neurofilament light chain (NfL) is released into extracellular fluids in response to axonal damage.^3,4^ Cerebrospinal fluid (CSF) NfL levels seem to reflect axonal damage in various neurological conditions, including multiple sclerosis and neurodegenerative dementias, and are therefore not specific for a certain disease.^5,6^ Nevertheless, NfL levels have been shown to differentiate sporadic PD from multiple system atrophy and progressive supranuclear palsy.^4,7,8^ Although 1 small study did not show a longitudinal change in NfL level during progression in PD,^9^ a recent single-center study on plasma NfL showed a modest correlation of plasma NfL with the Movement Disorder Society (MDS)–UPDRS III.^10^ Longitudinal analyses of NfL in larger multicenter cohorts, including prodromal and monogenetic participants, have not been carried out.

We tested the hypotheses that NfL would (1) show a positive correlation between serum and CSF values, (2) be higher in PD participants than in healthy controls (HCs), (3) be even greater in participants with other cognate or neurodegenerative disorders (ONDs), (4) increase over time with disease progression, (5) be higher in prodromal and asymptomatic mutation carriers than in controls, and (6) correlate with clinical measures and/or imaging indices of progression in a single-center cohort (DeNoPa) and a large multicenter cohort (PPMI).

## Methods

### Study Populations

#### The DeNoPa Cohort

As previously described,^11,12^ De Novo Parkinson’s disease (DeNoPa) is an ongoing prospective longitudinal, single-center study. NfL measurements were obtained in the CSF of 176 participants, including (1) newly diagnosed, drug-naive PD patients; (2) age-, sex-, and education-matched HCs; and (3) participants who were initially enrolled as having PD but on clinical follow-up had their diagnoses revised to a cognate or neurodegenerative disorder (OND; for details, see Supplement). Inclusion and exclusion criteria for the DeNoPa cohort including the identification of ONDs have been published previously and can be found in the Supplement.^11,12^ The number of participants in each group is given in Supplementary Table e1. The clinical assessment battery of the cohort has also been published previously.^11^ In brief, motor assessment used the revised Unified Parkinson’s Disease Rating Scale published by the Movement Disorder Society (MDS-UPDRS III and total score).^14^ Cognition was assessed using the Mini–Mental State Examination. The prespecified times for CSF collection were baseline and the 6-, 24-, 48-, and 72-month visits (Table 1).

To evaluate the possibility of testing more accessible serum samples in the larger Parkinson’s Progression Marker Initiative (PPMI) cohort, we next included a “bridging” cohort with paired CSF and serum samples that comprised 514 participants with PD and OND and healthy controls (Supplementary Table e2).

#### The PPMI Cohort

As previously described,^13^ the PPMI is an ongoing prospective longitudinal, observational, international multicenter study that aims to identify PD biomarkers (for inclusion criteria, see the Supplement). We retrieved the clinical data from the PPMI data portal at the Laboratory of Neuroimaging at the University of Southern California on June 6, 2019 (www.ppmi-info.org) and obtained all the available aliquots at prespecified times from the PPMI population. This generated serum NfL measurements of 1190 participants, including (1) newly diagnosed, drug-naive PD patients; (2) age- and sex-matched HCs: (3) prodromal participants with isolated rapid eye movement sleep behavior disorder (iRBD) or hyposmia; (4) the PPMI genetic cohort comprised of individuals with pathogenic gene mutations in *LRRK2, GBA*, or *SNCA*; and (5) participants initially enrolled as PD, but who had their diagnoses revised to OND on clinical follow-up. These included 4 patients with a clinical diagnosis according to atypical features indicative for multiple system atrophy (MSA), 1 with a clinical diagnosis of dementia with Lewy bodies (DLB), and 1 with clinical evidence for corticobasal degeneration. In addition to these 6 clinical diagnoses, 2 individuals died and were part of the brain donation program that confirmed the clinical diagnosis of MSA and DLB by immunohistochemistry. The number of participants in each group and the demographics and clinical data are in Table 1.

Participants with the following mutations were included in the analysis of the genetic cohort: *SNCA* rs356181, *SNCA* rs3910105, *LRRK2* G2019S rs34637584, *LRRK2* G2385R rs34778348, *LRRK2* R1441H rs34995376, *LRRK2* rs35801418, *LRRK2* I2020T rs35870237, *GBA* N370S rs76763715, *SREBF1* rs11868035, *MAPT* rs17649553.

The prespecified times for aliquot collection were baseline and the 6-, 12-, 24-, 36-, 48- and 60-month visits for PD participants and HCs and baseline and the 6-,12-, 24- and 36-month visits for prodromal and monogenetic participants. For the unaffected *GBA* mutation carriers in the genetic cohort, the available follow-up was until the 24-month visit.

The clinical assessment battery, mainly of the core cohort, is described on the PPMI website and has been published previously.^13^ In brief, motor assessment used the MDS-UPDRS III and total score. Use of medications for PD was recorded at each visit after baseline assessment and is expressed as levodopa-equivalent daily doses (LEDD).^15^

Cognitive testing included the Montreal Cognitive Assessment and psychometric tests of memory (Hopkins Verbal Learning Tests [HVLT]with immediate/total recall, discrimination recognition [HVLT-DG], and retention [HVLT-RT]), processing speed/attention (Symbol Digit Modality Test [SDMT]), executive function/working memory (WMS-III Letter-Number Sequencing test), and visuospatial abilities (Benton Judgment of Line Orientation test).^16^

Dopamine SPECT imaging was performed by dopamine transporter imaging (DaT) using standardized methods. Quantitative DaT measures of the striatal binding ratio of caudate, putamen, or striatal uptake were used in our analyses.

### Sample Processing and Immunoassays for NfL Quantification

NfL in CSF samples of the DeNoPa cohort was quantified by the commercial enzyme-linked immunosorbent assay (Uman-Diagnostics NfL ELISA kit; Umeå, Sweden),^17^ which has been validated in 6 different European laboratories.^18^ For all other samples (bridging cohort and PPMI), newly developed assays have been used and analyzed on a fully automated SIMOA HD-1 Analyzer (Quanterix, Lexington, MA). These assays have been shown to be up to 1000-fold more sensitive than conventional immunoassays with limits of quantification in the lower fg/mL range (also important for serum NfL measurements; for more information on validation and quality control, see Supplementary Material).^19^ Samples of the bridging cohort were analyzed in Kassel/Göttingen by a Simoa Neurology 4-Plex A Kit in duplicate, with the investigator blinded to the diagnosis. A Simoa NF-Light Advantage Kit was used for the serum samples of the PPMI cohort analyzed in duplicate at Quanterix (Lexington, MA), with the investigators and analysts blinded to the diagnosis. The slight discrepancy of means in the serum of the bridging and PPMI cohorts is because of the minimal differences between the 2 versions of the assays.

For the DeNoPa cohort (and the bridging cohort), all samples were collected in the morning under fasting conditions using Monovette tubes (Sarstedt, Nümbrecht, Germany) for serum collection by venipuncture. CSF was collected in polypropylene tubes (Sarstedt, Nümbrecht, Germany) directly after the serum collection by lumbar puncture in the sitting position. Tubes were centrifuged at 2500*g* at room temperature (20°C) for 10 minutes and aliquoted and frozen within 30 minutes after collection at −80°C until analysis. Before centrifugation white and red blood cell counts in CSF were determined manually.^11,20^

In the PPMI cohort serum was collected using standardized venipuncture procedures. Sample handling, shipment, and storage were carried out according to the PPMI biologics manual (http://ppmi-info.org). Aliquots of 0.5 mL of frozen serum were used for analysis.

### Standard Protocol Approvals, Registrations, and Patient Consent

Approval was received from the local ethical standards committee on human experimentation for all human participants in all cohorts (FF 89/2008, FF 130/2012, MC 310/2010). Written informed consent for research was obtained from all study participants. DeNoPa is registered in the German Register for Clinical trials (DRKS00000540), PPMI in clinicaltrials.gov as NCT01141023.

### Statistical Analysis

#### Analysis of the DeNoPa Cohort

The analysis of the DeNoPa cohort was carried out in a way identical to those carried out in the PPMI (described in more detail below), with a linear mixed model with log2NfL as response to longitudinal time and diagnostic groups. Because the DeNoPa cohort was age- and sex-matched, no adjustment was carried out. The Kruskal-Wallis test was used to determine if there were significant differences between more than 3 groups followed by pairwise comparisons using the Mann-Whitney *U* test adjusted using Tukeýs honestly significant difference.

The correlation of NfL in paired CSF and serum samples and the clinical measures in the bridging cohort were analyzed by Spearman’s rank test.

#### Analysis of the PPMI Cohort

Unlike the DeNoPa cohort, the PPMI cohort was not age- and sex-matched. We explored the association between log2NfL, age and sex among the HCs by locally estimated scatterplot smoothing, and a linear regression model. We used log transformation, as the distribution of NfL was right-skewed. To visualize the changes in NfL over time among different diagnostic groups, age- and sex-adjusted log2NfL was computed and plotted against the disease duration for patients with PD or OND and the time in the study for HCs, unaffected mutation carriers, and prodromal participants. Because of the skewed levels, we compared the median (instead of the mean) baseline NfL using a linear regression model. To compare changes over time, we fitted a linear mixed model for log2NfL with disease duration, diagnostic groups and their interactions as covariates, adjusted by age and sex. For HCs, unaffected mutation carriers, and prodromal participants, their disease durations were replaced by the time in the study.

Finally, we examined the association between the clinical measurements and NfL among patients with PD by fitting a linear median mixed model for each clinical measurement, with adjusted log2NfL, age, sex, disease duration, and whether the patient was on or off medication at the visit as the covariates.

The linear mixed models were fitted using the R package “lme4,” and the linear median mixed model was fitted using the R package “lqmm.” For all cohorts, the *P* values were adjusted via controlling the false discovery rate (FDR) using the Benjamini & Hochberg method.^21^

Linear models and linear fixed-effects models are only valid if the data used satisfy the underlying model assumptions. In particular, for all the statistical models used here, we tested the assumptions by plotting the residuals of the models against the fitted values and found no apparent pattern in the errors around 0. Hence, no existence of heteroscedastic (the variance of the residuals is not equal across groups) was observed. If the assumptions were violated, we opted for robust estimation using linear median mixed models.

## Results

### NfL Measures in the DeNoPa Cohort

**FIG. 1.**
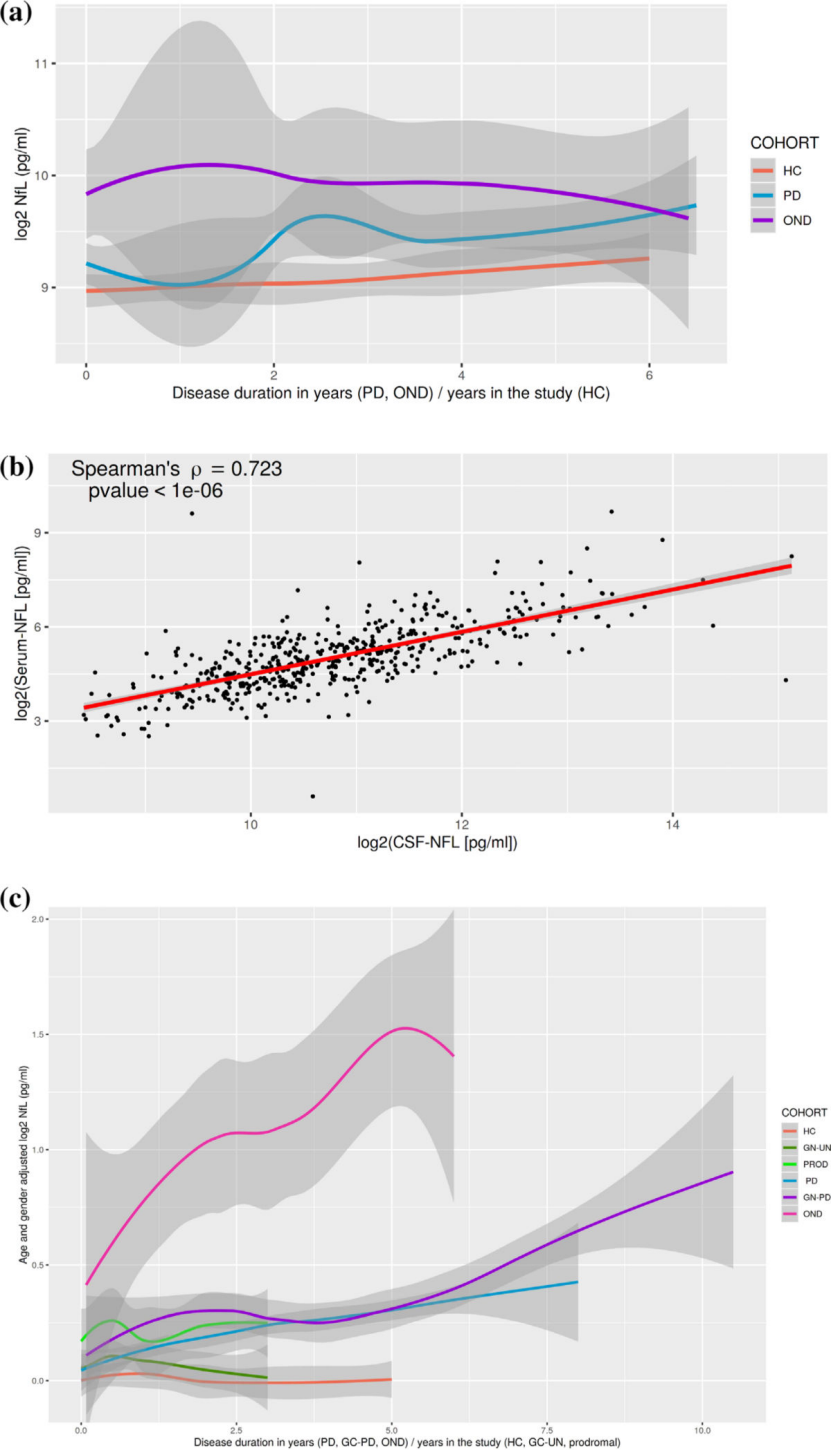
(**a**) Log2-transformed CSF NfL levels at each visit in healthy controls (HCs), Parkinson’s disease (PD), other neurodegenerative disorders (OND), in the DeNoPa cohort (demographics in Supplementary Table e1). The gray ribbon provides estimates of the standard error. (**b**) Spearman’s correlation of CSF and serum NfL in the bridging cohort (demographics are in Supplementary Table e3). (**c**) Age- and sex-adjusted log2-transformed serum NfL levels at each visit in HCs, Parkinson’s disease (PD), other neurodegenerative disorders (OND), prodromal with hyposmia or isolated REM sleep behavior disorder (PROD), and the genetic cohort with affected mutation carriers (GC-PD) and unaffected mutation carriers (GC-UN) in the PPMI cohort (demographics in Table 1). The gray ribbon provides estimates of the standard error. [Color figure can be viewed at wileyonlinelibrary.com]

Quantification of NfL was first carried out in CSF samples from the DeNoPa cohort. Supplementary Table e1 shows the baseline values of CSF NfL and clinical scores. Among the 176 participants, patients with OND had the highest baseline median CSF NfL at 839 pg/mL, followed by PD patients (562 pg/mL) and HCs (494 pg/mL), *P* = 0.01 (Fig. 1a). Linear mixed-effects model of log2NfL on CSF NfL diagnoses, time, and their interactions did not show significant differences across the groups in this small sample set (Supplementary Table e2).

In a second exploratory step, we analyzed a cross-sectional bridging cohort in which CSF and serum NfL levels were measured as a bridging step between the CSF samples measured in DeNoPa and the serum samples analyzed in PPMI (the cohort demographics are reported in Supplementary Table e2). The strong correlation between CSF and serum NfL (by Spearman’s rank P^=0.723; *P* <10 × 10^−6^; Fig. 1b) encouraged us to move to the validation step using serum samples from the longitudinal PPMI cohort.

### Serum NfL Measures in the PPMI Cohort

The baseline characteristics of the PPMI cohort (Table 1) show significant differences in age and sex across the cohorts. Within the HC group there is an age- and sex-dependent change in serum NfL. Linear regression of log2NfL with age and sex as covariates explained 51% of the NfL variability (*R*^2^ = 0.51; Supplementary Fig. 1), as has also been shown in other cohorts.^22^ The downstream analyses of the PPMI data were all age- and sex-adjusted. Patients with OND had the highest baseline age- and sex-adjusted median serum NfL (16.23 pg/mL), followed by patients with genetic PD (13.36 pg/mL), prodromal participants (12.20 pg/mL), PD patients (11.73 pg/mL), unaffected mutation carriers (11.63 pg/mL), and HCs (11.05 pg/mL); the *F* test of any differences among the medians had a *P* < 0.0001 (Fig. 1c).

Using a linear mixed model (Table 2), median serum NfL increased by 3.35% per year of age (*P* < 0.0001), and women had a median serum NfL level 6.79% higher than men (*P* = 0.0002).

With a linear median mixed model and adjustment for LEDD, when the adjusted NfL levels in serum doubled, the median MDS-UPDRS total score increased by 3.45 points (FDR-adjusted *P* = 0.0115), the median SDM total score decreased by 1.39 (FDR *P* = 0.026), the median HVLT-DG score decreased by 0.3 (FDR-adjusted *P* = 0.03), and the median HVLT-RT score decreased by 0.029 (FDR-adjusted *P* = 0.04). There was also a trend of increased MDS-UPDRS III with increased serum NfL (Table 3).

## Discussion

We investigated NfL levels in CSF in a longitudinal cohort (DeNoPa) from a single center and in serum in a longitudinal multicenter cohort (PPMI) consisting of HCs and prodromal and established PD participants. We also studied symptomatic and asymptomatic mutation carriers of known PD genetic mutations with follow-up of up to 6 years. We also explored the correlation between CSF and serum NfL levels to better understand the advantage of using a peripheral and less invasive source in which to measure NfL. Results obtained from the bridging cohort indicated that serum and CSF NfL levels were highest in OND and higher in PD participants compared with controls and that serum and CSF NfL levels correlated significantly. We are aware that the diagnostic accuracy of NfL in CSF and serum for PD patients versus HCs is small (with areas under the receiver operating curves around 0.6) and that NfL levels in persons with other cognate or neurodegenerative disorders are typically higher than in PD.

Importantly, the PPMI cohort comprises the largest multicenter NfL serum level data set reported to date. The main findings in the PPMI cohort were: (1) mean serum NfL was higher in established PD patients than HCs at each of the 6 times during the first 5 years after diagnosis, including at early stages of the disease; (2) markedly higher NfL was seen in the few participants with OND, including participants with dementia with Lewy bodies (DLB) and multiple system atrophy (MSA), discussed in more detail below; (3) there was a significant (age-adjusted) longitudinal increase in serum NfL in PD patients compared with HCs; and (4) the longitudinal change in serum NfL correlated significantly with MDS-UPDRS total score and some cognitive measures, suggesting that serum NfL may be a biomarker of clinical progression (independent of age and sex). Overall, the longitudinal analysis of serum NfL in PPMI confirmed the findings from DeNoPa. The PPMI cohort enabled us to analyze prodromal conditions and a genetic cohort with symptomatic and asymptomatic mutation carriers. We found that the serum NfL levels of participants with 2 prodromal conditions for α-synuclein aggregation disorders had higher levels than PD patients or HCs and lower than patients with ONDs, including MSA and DLB. This was as expected because iRBD participants in particular have a high risk of predominantly converting to PD as well as to DLB and MSA. The 17 MSA cases in the OND group of the cross-sectional discovery cohort showed the highest median values in CSF (2829.86 pg/mL; range, 794.32–6015.71 pg/mL) and in serum (37.42 pg/mL; range, 21.26–91.99 pg/mL). In the genetic cohort, serum NfL levels of asymptomatic mutation carriers in all 3 mutation carrier groups were lower than in their respective symptomatic group. The levels in all 6 genetic groups (symptomatic and asymptomatic) remained relatively stable over the 2 to 3 years of follow-up, and there were no differences in NfL rate of change between the mutation carrier groups. Subsequent analyses will explore the effects of disease duration on changes in serum NfL.

Elevated levels of NfL, as seen here in participants with PD and OND compared with HCs, have been identified in several other neurological conditions including dementia disorders and multiple sclerosis.^22^ Therefore, this marker for axonal damage is not specific for any disease,^4^ but could be useful for exploring specific questions within disease entities. Within PD spectrum disorders, NfL may be particularly useful in discriminating PD from cognate disorders such as MSA, progressive supranuclear palsy (PSP), and DLB, as has been previously described.^7^ Because of the high rate of misdiagnoses in early PD, as shown in a neuropathologically confirmed cohort,^2^ there is a need for biomarkers to distinguish sporadic PD from other neurodegenerative disorders with parkinsonian syndromes, such as the 2 α-synuclein aggregation disorders, DLB with early dementia and marked hallucinations and MSA with clinically severe autonomic disturbances and α-synuclein deposition in the glia (and not in neurons), thus disorders with a completely different clinical and neuropathological disease phenotype. This is important in clinical practice as “Parkinson-plus” syndromes may have a completely different underlying pathophysiology, have worse prognoses, and may ultimately require different therapy regimens. This need will grow as medicine becomes more personalized. In addition, the ability to select patients for inclusion or stratification within clinical trials is also becoming increasingly important. The diagnosis of PD in PPMI is clinical; it is based on systematic history and examination, structural MRI (to rule out other diseases), pathological DaT by central read, and most importantly by longitudinal clinical follow-up. Based on this process, 8 participants in the PD cohort analyzed here were found to have diagnoses other than PD after 5 years of follow-up and were thus taken out of the PD cohort. This group of participants, despite the small size, was separated in the analysis as OND. Serum NfL levels in this OND group were higher at baseline (before the follow-up and before evolving into another disease) and may provide future diagnostic utility. Additional studies including analysis on samples from postmortem PD-confirmed participants will further establish the utility of serum NfL measurements in differentiating PD from OND. In numerous previous CSF studies (conducted before ultrasensitive technologies were available), the mean NfL levels in OND were found to be markedly increased compared with HCs, such as in multiple sclerosis (4.5-fold increase), traumatic brain injury (3-fold increase), PSP, corticobasal degeneration, MSA (3- to 4.25-fold increase) as well as in other, more slowly progressing neurodegenerative disorders such as Alzheimer’s disease (1.5- to 2-fold increase).^22^ Compared with these disorders, the increase is smaller in PD (1.25 in CSF and 1.4 in serum). Two reasons for this smaller increase in PD are possible: (1) PD progresses more slowly than other neurological disorders that have more severe damage to myelinated tracts (as seen in multiple sclerosis), or (2) there is less cell death compared with, for example, Alzheimer’s disease or Creutzfeldt-Jakob disease, in which there is even more extreme cell death. It is known that in PD α-synuclein aggregation occurs mainly in neurons with high energy turnover in less myelinated axons,^23^ whereas NfL is mainly expressed in larger myelinated axons.^24^ Thus, our data are consistent with the concept that increases in NfL are not disease specific, but reflect the magnitude and perhaps the tempo of neuronal damage of larger myelinated axons in any disease entity. Also, in contrast to many more rapidly progressive or acute diseases, the pathological changes in PD and the respective cell loss are only mild.^25^

Despite the slight increase shown here, the relatively higher serum NfL in the prodromal groups (compared with PD patients and HCs), especially in the iRBD group, may indicate the presence of active disease and the potential for conversion to either PD or parkinsonian syndromes. Furthermore, in fact, longitudinal NfL levels may be highest in the early stages of the disease with the greatest disease activity, as has been similarly seen in β-amyloid in Alzheimer’s disease.^26^ NfL levels are also higher in presymptomatic cases with amyotrophic lateral sclerosis.^27^

Another variable affecting the increase of NfL levels is age. Across published studies, NfL in CSF and blood increases with age,^22^ as we also identified in the PPMI cohort. For example, the prodromal groups in PPMI are on average 3–5 years older than the PD group and the iRBD participants are older than the hyposmic participants featuring higher NfL values. This positive association of serum NfL and age is explained by structural alterations of the axons with aging, including vascular disease, metabolic changes, and inflammation. All these have also been shown to play a role in PD progression,^28^ which may also influence NfL levels in blood. To account for these systematic differences at baseline, we adjusted all our serum analyses for age and sex.

Similar to a previous report showing that CSF NfL levels were relatively stable despite disease progression in PD over 12 months,^9^ we observed a slight increase in serum NfL over 72 months of follow-up, which did not correlate with motor progression. The discrepancy between CSF and serum measurements could possibly be because of the smaller CSF sample size.

In contrast, NfL levels in serum increased significantly over a 60-month follow-up in the PPMI cohort. The longitudinal increase in the age- and sex-adjusted serum NfL significantly correlated with changes over time in MDS-UPDRS total score in the PPMI cohort. A significant association with longitudinal progression of age- and sex-adjusted serum NfL was also seen for the Hopkins Verbal Learning Test discrimination recognition and retention as well as for processing the speed/attention test SDMT.

We did not observe a longitudinal change in serum NfL in the prodromal cohort. This might be because of small sample size and the heterogeneity of the group as recruited, both in terms of disease stage and as an eventual diagnostic category and phenotypes of individual participants. The apparent stability over time in the prodromal cohort could also indicate that serum NfL levels do not change continuously before motor symptoms evolve. This suggestion is also supported by the longitudinal stability of serum NfL in the asymptomatic mutation carriers. Only continued follow-up will identify participants converting to a motor disease or a symptomatic disease state. The individual levels may indicate the prognostic direction.

The strengths of our study are the 2 different independent longitudinal cohorts, especially the large PPMI cohort including prodromal subjects and asymptomatic mutation carriers and the longitudinal times of up to a 60-month follow-up sampling. Limitations are the smaller CSF sample size in DeNoPa and the variability of the data, indicating heterogeneity of disease that is well known and discussed. The NfL data from PPMI are available online and will be strengthened with continued analyses of these data using additional alternative statistical models as well as follow-up of the cohorts, especially in participants at risk for disease progression. In addition, monitoring of larger longitudinal cohorts with a focus on prodromal or asymptomatic PD with longer observational time to allow the development of motor disease are needed and will be pursued in the extended PPMI 2.0 starting in 2020. This being said, we remain cognizant that increased NfL levels reflect neuronal and axonal damage and are not specific to PD or any other neurodegenerative disorder. Thus, more specific markers will need to be identified, leading hopefully to a panel of different markers reflecting disease state, rate, and fate. Finally, we note the profound influence of age and sex on serum NfL levels. Therefore, we recommend that age- and sex-based adjustments be applied when interpreting serum NfL levels in clinical research and practice.

## Supplementary Material

Supplementary Material

Photo

## Figures and Tables

**TABLE 1.** Demographics, clinical, dopamine transporter imaging, and baseline serum NfL data in the PPMI cohort

Parameter	Level	Healthy controls (HCs)	Parkinson’s disease (PD) patients	Other neurodegenerative diseases (OND)	Prodromal (PROD)	Genetic cohort - PD (GN-PD)	Genetic cohort - unaffected (GN-UN)	FDR-adjusted P value across all groups

n		187	397	8	63	226	309	
Sex	Female	67 (35.8%)	140 (35.3%)	2 (25.0%)	14 (22.2%)	117 (51.8%)	183 (59.2%)	< 0.0^s^
	Male	120 (64.2%)	257 (64.7%)	6 (75.0%)	49 (77.8%)	109 (48.2%)	126 (40.8%)	
Age	Mean ± SD	61 ± 11	62 ± 9.9	64 ± 6.8	69 ± 5.8	62 ± 10	62 ± 7.4	< 0.0^b^
	Median (min, max)	62 (31, 84)	62 (34, 85)	66 (53, 73)	67 (59, 83)	65 (32, 85)	62 (34, 84)	
MDS-UPDRS	Mean ± SD	1.2 ± 2.2	21 ± 8.9	27 ± 5.4	3.9 ± 3.8	23 ± 11	2.7 ± 3.9	< 0.0^b^
part III	Median (min, max)	0 (0, 13)	20 (4, 51)	28 (18, 36)	3 (0, 15)	21 (4, 56)	1 (0, 27)	
MDS-UPDRS Total Score	Mean ± SD	4.6 ± 4.4	32 ± 13	47 ± 10	12 ± 7.8	38 ± 18	8.6 ± 7.7	< 0.0^b^
	Median (min, max)	3 (0, 20)	31 (7, 70)	44 (37, 68)	11 (0, 31)	36 (7, 113)	7 (0, 45)	
MoCA	Mean ± SD	28 ± 1.1	27 ± 2.3	27 ± 2.3	26 ± 3.5	26 ± 3.5	27 ± 2.3	< 0.0^b^
	Median (min, max)	28 (27, 30)	28 (17, 30)	28 (23, 30)	27 (11, 30)	27 (11, 30)	27 (16, 30)	
HVLT-IR	Mean ± SD	26 ± 4.5	24 ± 5	26 ± 4.6	22 ± 5.3	24 ± 5.6	27 ± 4.9	< 0.0^b^
	Median (min, max)	26 (15, 35)	25 (9, 36)	25 (18, 32)	21 (9, 33)	24 (7, 34)	27 (9, 36)	
HVLT-DG	Mean ± SD	10 ± 2.8	9.7 ± 2.6	9.9 ± 1.7	9.4 ± 2.2	9.9 ± 2.3	11 ± 1.8	< 0.0^b^
	Median (min, max)	11 (−4, 12)	10 (−4, 12)	10 (7, 12)	10 (2, 12)	10 (−1, 12)	11 (0, 12)	
HVLT-RT	Mean ± SD	0.9 ± 0.18	0.85 ± 0.2	0.86 ± 0.17	0.77 ± 0.29	0.8 ± 0.25	0.88 ± 0.17	< 0.0^b^
	Median (min, max)	0.92 (0.27, 1.5)	0.9 (0, 1.3)	0.8 (0.71, 1.2)	0.83 (0, 1.2)	0.89 (0, 1.4)	0.92 (0.12, 1.4)	
SDMT	Mean ± SD	47 ± 11	41 ± 9.8	38 ± 7.6	36 ± 11	38 ± 12	46 ± 9.3	< 0.0^b^
	Median (min, max)	46 (20, 83)	42 (7, 82)	40 (23, 46)	35 (15, 56)	39 (6, 75)	47 (14, 74)	
LNS	Mean ± SD	11 ± 2.6	11 ± 2.7	10 ± 1.4	9.4 ± 2.9	9.6 ± 3.1	11 ± 2.8	< 0.0^b^
	Median (min, max)	11 (2, 20)	11 (2, 20)	10 (9, 13)	9 (3, 17)	10 (2, 18)	11 (0, 20)	
BJLO	Mean ± SD	13 ± 2	13 ± 2.1	13 ± 2.1	12 ± 2.3	12 ± 3	13 ± 2.1	< 0.0^b^
	Median (min, max)	14 (4, 15)	13 (5, 15)	13 (8, 15)	12 (3, 15)	12 (0, 15)	13 (1, 15)	
DaT scan putamen (SBR)	Mea n± SD	2.1± 0.54	0.82 ± 0.28	0.63 ± 0.32	NA	0.76 ± 0.35	NA	< 0.0^b^
	Median (min, max)	2.1 (0.64, 3.9)	0.79 (0.24, 2.2)	0.55 (0.31, 1.3)	NA	0.7 (0.21, 2.4)	NA	
DaT scan caudate (SBR)	Mean ± SD	3 ± 0.6	2 ± 0.54	1.4 ± 0.64	NA	1.8 ± 0.6	NA	< 0.0^b^
	Median (min, max)	2.9 (1.3, 4.8)	2 (0.39, 3.7)	1.2 (0.74, 2.6)	NA	1.8 (0.43, 3.9)	NA	
DaT scan striatum (SBR)	Mean ± SD	2.6± 0.55	1.4 ± 0.39	1 ± 0.47	NA	1.3 ± 0.45	NA	< 0.0^b^
	Median (min, max)	2.4 (0.98, 4.2)	1.4 (0.31, 2.6)	0.91 (0.53, 1.9)	NA	1.3 (0.38, 3.1)	NA	
Serum NfL (pg/mL)	Mean ± SD Median (min, max)	12 ± 6.7 10 (2.4, 51)	13 ± 7.2 11 (1.8, 77)	18 ± 7 14 (12, 29)	16 ± 7 14 (6.5, 39)	15 ± 10 13 (3.8, 81)	13 ± 6.7 11 (2, 63)	< 0.0^b^

MoCA, education-adjusted Montreal Cognitive Assessment; SDMT, Symbol Digit Modality Test; WMS-III, executive function/working memory; LNS, Letter-Number Sequencing Test; BJLO, Benton Judgment of Line Orientation test; MDS-UPDRS, Movement Disorder Society Unified Parkinson’s Disease Rating Scale; DaT scan, dopamine transporter imaging; SBR, striatal binding ratio; NfL, neurofilament light chain; HVLT-IR, Hopkins Verbal Learning Tests with immediate/total recall; HVLT-DG, Hopkins Verbal Learning Tests wwith discrimination recognition; HVLT-RT, Hopkins Verbal Learning Tests with retention; SSD, sandard deviation.

a Pearson’s chi-square test.

b Kruskal-Wallis rank sum test

**TABLE 2.** Linear mixed-effects model of log2NfL on diagnoses, time, and their interactions, adjusted by age and sex

Variable	Estimate	2^estimate^	Standard error	*p*

(Intercept)	0.5636	1.4780	0.1017	**< 0.0001**
Age	0.0475	1.0335	0.0015	**< 0.0001**
Sex: male	−0.0947	0.9364	0.0299	**0.0016**
Diagnostic group: genetic cohort — unaffected	0.0774	1.0551	0.0494	0.1178
Diagnostic goup: prodromal	0.1613	1.1183	0.0781	**0.0392**
Diagnostic group: Parkinson’s disease (PD)	0.0615	1.0435	0.0466	0.1874
Diagnostic group: genetic cohort — PD	0.1966	1.1460	0.0650	**0.0025**
Diagnostic group: other neurodegenerative diseases (OND)	0.5074	1.4215	0.1927	**0.0086**
Time	0.0008	1.0005	0.0094	0.9360
Diagnostic group: genetic cohort — unaffected × time	0.0039	1.0027	0.0175	0.8232
Diagnostic group: prodromal × time	0.0123	1.0085	0.0235	0.6022
Diagnostic group: Parkinson’s disease × time	0.0541	1.0382	0.0111	**< 0.0001**
Diagnostic group: genetic cohort — PD × time	0.0358	1.0251	0.0150	**0.0175**
Diagnostic group: other neurodegenerative diseases × time	0.2165	1.1619	0.0435	**< 0.0001**

**TABLE 3.** Associations between clinical measurements and age-, sex-, and levodopa-equivalent dosages adjusted log2NfL among patients with Parkinson’s disease in the PPMI cohort

Variable	Estimate	Standard error	Lower bound	Upper bound	*P*	FDR-adjusted *P*

MDS-UPDRS part III	1.3850	0.6214	0.1360	2.6340	0.0305	0.0731
MDS-UPDRS total score	3.4450	0.9802	1.4760	5.4150	0.0010	**0.0115**
MoCA	−0.4153	0.2412	−0.9001	0.0695	0.0915	0.1568
HVLT-IR	−0.6134	0.3790	−1.3750	0.1482	0.1120	0.1680
HVLT-DG	−0.3019	0.1100	−0.5230	−0.0807	0.0085	**0.0339**
HVLT-RT	−0.0286	0.0112	−0.0512	−0.0061	0.0138	**0.0415**
SDMT	−1.3930	0.4657	−2.3280	−0.4567	0.0044	**0.0261**
LNS	−0.0683	0.1339	−0.3373	0.2007	0.6123	0.6679
BJLO	−0.2431	0.1404	−0.5253	0.0390	0.0896	0.1568
DaT scan putamen (SBR)	−0.0244	0.0253	−0.0752	0.0265	0.3398	0.4531
DaT scan caudate (SBR)	−0.0225	0.0420	−0.1069	0.0618	0.5937	0.6679
DaT scan striatum (SBR)	0.0038	0.0400	−0.0766	0.0841	0.9254	0.9254

MoCA, education-adjusted Montreal Cognitive Assessment; SDMT, Symbol Digit Modality Test; LNS, Letter-Number Sequencing Test; BJLQ, Benton Judgment of Line Orientation test; MDS-UPDRS, Movement Disorder Society Unified Parkinson’s Disease Rating Scale; DaT scan, dopamine transporter imaging; SBR, striatal binding ratio; HVLT-IR, Hopkins Verbal Learning Tests with immediate/total recall; HVLT-DG, Hopkins Verbal Learning Tests with discrimination recognition; HVLT-RT, Hopkins Verbal Learning Tests with retention (HVLT-RT).
