# Supplementary material for: Validation of Serum Neurofilament Light Chain as a Biomarker of Parkinson’s Disease Progression
Source: Mov Disord. Author manuscript; Available in PMC 2021 Apr 2. (PMC8017468; doi:10.1002/mds.28206)
Supplement: Supplementary Material [file NIHMS1682170-supplement-Supplementary_Material.docx]

**Supplementary material: Validation of Serum Neurofilament Light Chain as a Biomarker of Parkinson’s Disease Progression**

**Methods**

**The DeNoPacohort inclusion criteria**

Between 2009 and 2012 we enrolled 159 subjects with Parkinson’s disease (PD) and 110 healthy controls (HC) at the Paracelsus-Elena-Klinik, Kassel, Germany.[^1^](#_ENREF_1) Detailed inclusion criteria have been described previously.[^1^](#_ENREF_1) Briefly, subjects had to be between 40 and 85 years old, newly diagnosed with PD featuring at least two of resting tremor, bradykinesia, and rigidity according to UK Brain bank Criteria, and had to fulfill de novo PD criteria. Subjects with known severe vascular encephalopathy, normal-pressure hydrocephalus or signs or symptoms according to multiple system atrophy or progressive supranuclear palsy (according to consensus criteria) at baseline were excluded. Healthy controls had to be between 40 and 85 years old, without any active known/treated condition of the central nervous system and without a family history of idiopathic PD. Controls were matched using frequency matching by age, sex, and education. Written informed consent was obtained from each participant and our study was approved by the local ethics committees. The clinical diagnosis was reassessed at the second visit after 24 months by two teams of independent neurologists. The reassessment of clinical diagnosis revealed other neurological disorders in 17 of the patients: Three were classified as suffering from progressive supranuclear palsy, two from multiple system atrophy (MSA-P), three from dementia with Lewy Bodies, one from vascular parkinsonism, and in five cases the predominant movement abnormality was classified as essential tremor (n=3) or as cerebellar tremor (n=2). In three patients the final diagnosis remained unclear. One patient with MSA-P died after the 24-month follow-up visit, and autopsy confirmed the diagnosis of MSA-P.

**The bridging-cohort inclusion criteria**

CSF and serum samples from the cross-sectional trainings cohort were collected from in-patients with careful clinical phenotyping that included magnetic resonance imaging (MRI) to determine structural abnormalities, quantitative levodopa testing as published,[^15^](#_ENREF_15) smell identification test, MMSE followed by further cognitive testing and video-supported polysomnography (vPSG) to determine REM sleep behavior disorder (RBD) in a subset of patients. The phenotyping was undertaken in accordance with established criteria for PD, multiple system atrophy (MSA), dementia with Lewy bodies (DLB), progressive supranuclear palsy (PSP), corticobasal degeneration (CBD) and frontotemporal dementia (FTD). Participants with marked vascular lesions in 1.5 tesla magnetic resonance imaging (MRI) indicative for a vascular comorbidity and participants with normal pressure hydrocephalus by MRI were excluded.

**The PPMI-cohort inclusion criteria**

In PPMI the inclusion criteria for PD participants were the following: (1) aged over 30 years; (2) presence of two of the following: bradykinesia, rigidity and resting tremor, or an asymmetric resting tremor, or asymmetric bradykinesia; (3) diagnosis made within the last 24 months; (4) PD drug-naivety, and (5) dopamine transporter deficit in the putamen on the 123-I Ioflupane dopamine transporter imaging (DaT) by central reading. Isolated RBD participants met the following criteria: (1) men or women aged over 60 years, and (2) confirmation of RBD by video supported polysomnography (vPSG) with central reading and/or clinical diagnosis of RBD by site investigator including existing PSG as described. The central vPSG interpretation was based on the following criteria: (i) 18% of any EMG activity in m. mentalis, 32% of any EMG activity in mentalis and flexor digitorum superficialis (FDS) (in 3s bins), (ii) 27% of any EMG activity in m. mentalis, 32% of any EMG activity in m. mentalis and FDS (in 30s bins). In two cases a central PSG reading was not available due to technical difficulties with electronic PSG transfer, but these participants had a clinical diagnosis of isolated RBD (iRBD) by site investigator including prior PSG and also had to show decreased dopamine transporter imaging. Hyposmic participants were 60 years or older with olfaction at or below the 10th percentile by age and sex as determined by the University of Pennsylvania Smell Identification Test (UPSIT). All participants with iRBD and hyposmic participants also required confirmation from the imaging core at the Institute for Neurodegenerative Disorders (IND) that screening by DaT (or V-MAT-2-PET scan for sites where DaT is not available) was read as eligible. About 80% of the prodromal participants were selected with DaT deficit similar to participants with early PD, and 20% were selected with no DaT deficit. Prodromal participants without DaT deficit were similar in age, sex, and risk profile to those with mild to moderate dopamine transporter deficit. Exclusion criteria can be found in the study protocol at http://www.ppmi-info.org/study-design/research-documents-and-sops/.

**Validation and quality control of NfL measurements**

The Simoa assays have been shown to be up to 1,000-fold more sensitive than conventional immunoassays with limits of quantification in lower fg/ml range (important also for serum NfL measurements).[^20^](#_ENREF_20) For validation purposes we analyzed four CSF samples and three serum samples, diluted 1 in 100 to 1 in 800 and 1 in 4 to 1 in 32, respectively. In addition, recovery rates were determined in two spiked and non-spiked CSF and serum samples. NfL was detectable in all CSF and serum samples. Parallelism was observed for all CSF dilutions except for one sample diluted 1 in 800. For serum samples we observed parallelism for all samples except for one sample diluted 1 in 16 and one sample diluted 1 in 32. Recovery rates were more than 90% for CSF samples. Probably due to matrix effects, recovery rates were below 50% for serum samples. Here small amounts of the interphase generated during sample centrifugation were probably transferred to the assay plate. A direct comparison of CSF and serum samples on the single- and the multiplex assays showed that the measurements in single-versus multiplex assay are very comparable in levels.

The NF-light® assay (Simoa NF-Light Advantage Kit; Quanterix, Lexington, USA) was used for the serum samples of the PPMI cohort diluted 1:4 and analyzed in duplicates at Quanterix (Lexington, MA, USA) with the investigators and analysts being blinded to the diagnosis. Three sets of endogenous quality controls were run on all plates. Three sets of spiked quality controls (QCs; recombinant neurofilament spiked into sample matrix) were prepared in bulk and run on all plates. Analytical assay parameters (i.e. quality control sample acceptance ranges) were determined prior to sample testing.

**Supplementary figure 1:** Linear regression of baseline log2NfL on baseline age by gender among healthy controls in the PPMI-cohort.

**Supplementary table e1: Demographics and NfL measures in CSF of the DeNoPa-cohort**

| Parameter | Level | Healthy Control (HC) | | Parkinson's Disease (PD) | | Other Neurodegenerative Diseases (OND) | | FDR adjusted p- values (across all groups) |
| --- | --- | --- | --- | --- | --- | --- | --- | --- |
| **N** |  | 61 | | 98 | | 17 | |  |
| **Sex** |  |  | |  | |  | |  |
|  | Female | 20 (32.8%) | | 33 (33.7%) | | 4 (23.5%) | | 0.81^1^ |
|  | Male | 41 (67.2%) | | 65 (66.3%) | | 13 (76.5%) | |  |
| **Age at baseline** | |  | |  | |  | |  |
|  | mean±SD | 65 ± 6.8 | | 65 ± 9.9 | | 67 ± 7.6 | | 0.81^2^ |
|  | median (min; max) | 66 (44; 84) | | 66 (40; 84) | | 66 (53; 78) | |  |
| **MDS-UPDRS part III** | |  | |  | |  | |  |
|  | mean±SD | 0.62 ± 1.4 | | 23 ± 11 | | 26 ± 13 | | < 0.01^2^ |
|  | median (min; max) | 0 (0; 6) | | 22 (3; 54) | | 24 (6; 48) | |  |
| **MDS-UPDRS total score** | |  | |  | |  | |  |
|  | mean±SD | 3.3 ± 3.3 | | 36 ± 17 | | 43 ± 19 | | < 0.01^2^ |
|  | median (min; max) | 2 (0; 15) | | 36 (7; 84) | | 43 (14; 74) | |  |
| **MMSE total score** | |  | |  | |  | |  |
|  | mean±SD | 29 ± 1.2 | | 28 ± 1.4 | | 27 ± 2.4 | | 0.05^1^ |
|  | median (min; max) | 29 (26; 30) | | 29 (22; 30) | | 28 (21; 30) | |  |
| **Baseline** |  | |  | |  | |  |  |
| N |  | | 61 | | 98 | | 17 |  |
| **CSF NfL [pg/ml]** | | |  | |  | |  |  |
|  | mean±SD | | 543 ± 250 | | 675 ± 384 | | 1063 ± 670 | < 0.01^2^ |
|  | median (min; max) | | 494  (249; 1626) | | 562  (170; 2477) | | 830  (416; 2876) |  |
|  | Missing | | 9 | | 19 | | 0 |  |
| **24 months Follow-up** | | |  | |  | |  |  |
| N |  | | 60 | | 88 | | 18 |  |
| **CSF NfL [pg/ml]** | | |  | |  | |  |  |
|  | mean±SD | | 566 ± 261 | | 1170 ± 2486 | | 1240 ± 987 | < 0.01^2^ |
|  | median (min; max) | | 515  (259; 1618) | | 669  (169; 16449) | | 887  (519; 3878) |  |
|  | Missing | | 28 | | 45 | | 6 |  |
| **48 months** | **Follow-up** | |  | |  | |  |  |
| N |  | | 56 | | 85 | | 17 |  |
| **CSF NfL [pg/ml]** | | |  | |  | |  |  |
|  | mean±SD | | 611 ± 290 | | 751 ± 363 | | 1025 ± 476 | 0.02^2^ |
|  | median (min; max) | | 531  (271; 1630) | | 688  (201; 1869) | | 984  (426; 1677) |  |
|  | Missing | | 29 | | 42 | | 10 |  |
| **72 Months Follow-up** | | |  | |  | |  |  |
| N |  | | 53 | | 76 | | 17 |  |
| **CSF NfL [pg/ml]** | | |  | |  | |  |  |
|  | mean±SD | | 643 ± 228 | | 977 ± 903 | | 1016 ± 532 | 0.03^2^ |
|  | median (min; max) | | 605  (395; 1384) | | 762  (295; 5298) | | 888  (582; 1938) |  |
|  | Missing | | 32 | | 48 | | 12 |  |

^1^ Pearson's Chi-squared test

^2^ Kruskal-Wallis rank sum test

**Abbreviations:** Movement Disorders Society Unified Parkinson’s Disease rating Scale (MDS-UPDRS); Neurofilament light chain (NfL); Standard deviation (SD)

* At 24 months follow-up all participants were reassessed as to the differential diagnosis (by clinical history, response to levodopa, and neurological examination) 17 returning disease participants were diagnosed with a different disease including atypical PD (as published[^12^](#_ENREF_12)): PSP (n=3); MSA (n=1), dementia with Lewy Bodies (DLB; n=2), one each with dystonic and cerebellar tremor and in nine cases the diagnosis remained unclear but suggested a progressive neurodegenerative disorder. This group was separated from the PD group and classified as other neurodegenerative disorders (OND).

**Supplementary table e2**: Linear mixed effect model of log2NfL in CSF on diagnoses, time and their interactions, in the DeNoPa-cohort

| Variable | Estimate | 2^Estimate | Std. Error | p-value |
| --- | --- | --- | --- | --- |
| **(Intercept)** | 6.3500 | 81.5713 | 0.3669 | **<0.0001** |
| **Age** | 0.0366 | 1.0257 | 0.0053 | **<0.0001** |
| **Sex: male** | 0.3058 | 1.2361 | 0.0993 | **0.0024** |
| **Diagnostic group: Parkinson's Disease (PD)** | 0.3292 | 1.2563 | 0.1036 | **0.0018** |
| **Diagnostic group: Other Neurodegenerative Diseases (OND)** | 0.8064 | 1.7489 | 0.1700 | **<0.0001** |
| **Time** | 0.0189 | 1.0132 | 0.0168 | 0.2615 |
| **Diagnostic group: Parkinson's Disease*TIME** | 0.0261 | 1.0182 | 0.0209 | 0.2142 |
| **Diagnostic group: Other Neurodegenerative Diseases*TIME** | -0.0350 | 0.9760 | 0.0336 | 0.2986 |

**Supplementary table e3:** Demographics and NfL measures in CSF and serum in bridging-cohort

| 1. Parameter | 1. Level | 1. Healthy Control 2. (HC) | 1. Parkinson's Disease (PD) | 1. Other Neurodegenerative Diseases (OND) | 1. FDR adjusted p-value 2. across all groups |
| --- | --- | --- | --- | --- | --- |
| 1. **N** |  | 1. 20 | 1. 150 | 1. 344 |  |
| 1. **Sex** | 1. Male | 1. 14 (70.0%) | 1. 100 (66.7%) | 1. 224 (65.1%) | 1. 0.91^1^ |
|  | 1. Female | 1. 6 (30.0%) | 1. 50 (33.3%) | 1. 120 (34.9%) |  |
|  | 1. Missing | 1. 0 | 1. 0 | 1. 0 |  |
| 1. **Age** | 1. mean±SD | 1. 69 ± 6.4 | 1. 69 ± 9.6 | 1. 71 ± 8.7 | 1. 0.09^2^ |
|  | 1. median (min; max) | 1. 70 (57; 86) | 1. 71 (40; 86) | 1. 72 (34; 88) |  |
|  | 1. missing | 1. 0 | 1. 0 | 1. 1 |  |
| **UPDRS part III** | 1. mean±SD | 1. 1.7 ± 2.2 | 1. 31 ± 15 | 1. 30 ± 14 | 1. < 0.01^2^ |
|  | 1. median (min; max) | 1. 1 (0; 7) | 1. 30 (0; 75) | 1. 28 (1; 83) |  |
|  | 1. missing | 1. 0 | 1. 13 | 1. 84 |  |
| **UPDRS total score** | 1. mean±SD | 1. 4.2 ± 5.9 | 1. 55 ± 24 | 1. 53 ± 23 | 1. < 0.01^2^ |
|  | 1. median (min; max) | 1. 3 (0; 25) | 1. 54 (7; 135) | 1. 50 (5; 135) |  |
|  | 1. missing | 1. 0 | 1. 35 | 1. 175 |  |
| 1. **CSF NfL [pg/ml]** | 1. mean±SD | 1. 922 ± 446 | 1. 2124 ± 2180 | 1. 2779 ± 3544 | 1. < 0.01^2^ |
|  | 1. median (min; max) | 1. 752 2. (374; 2267) | 1. 1438 2. (342; 15304) | 1. 1895 2. (346; 35742) |  |
|  | 1. missing | 1. 0 | 1. 1 | 1. 3 |  |
| 1. **Serum NfL [pg/ml]** | 1. mean±SD | 1. 18 ± 8.4 | 1. 55 ± 105 | 1. 44 ± 38 | 1. < 0.01^2^ |
|  | 1. median (min; max) | 1. 15 2. (5.8; 43) | 1. 29 2. (1.5; 815) | 1. 33 2. (5.7; 304) |  |
|  | 1. missing | 1. 0 | 1. 1 | 1. 3 |  |

^1^ Fisher's Exact Test for count data

^2^ Kruskal-Wallis rank sum test

**Abbreviations:** Unified Parkinson’s Disease rating Scale (UPDRS); Neurofilament light chain (NfL); Cerebrospinal fluid (CSF)
