# Supplementary figures and images for: Validation of Serum Neurofilament Light Chain as a Biomarker of Parkinson’s Disease Progression
Source: Mov Disord. Author manuscript; Available in PMC 2021 Apr 2. (PMC8017468; doi:10.1002/mds.28206)

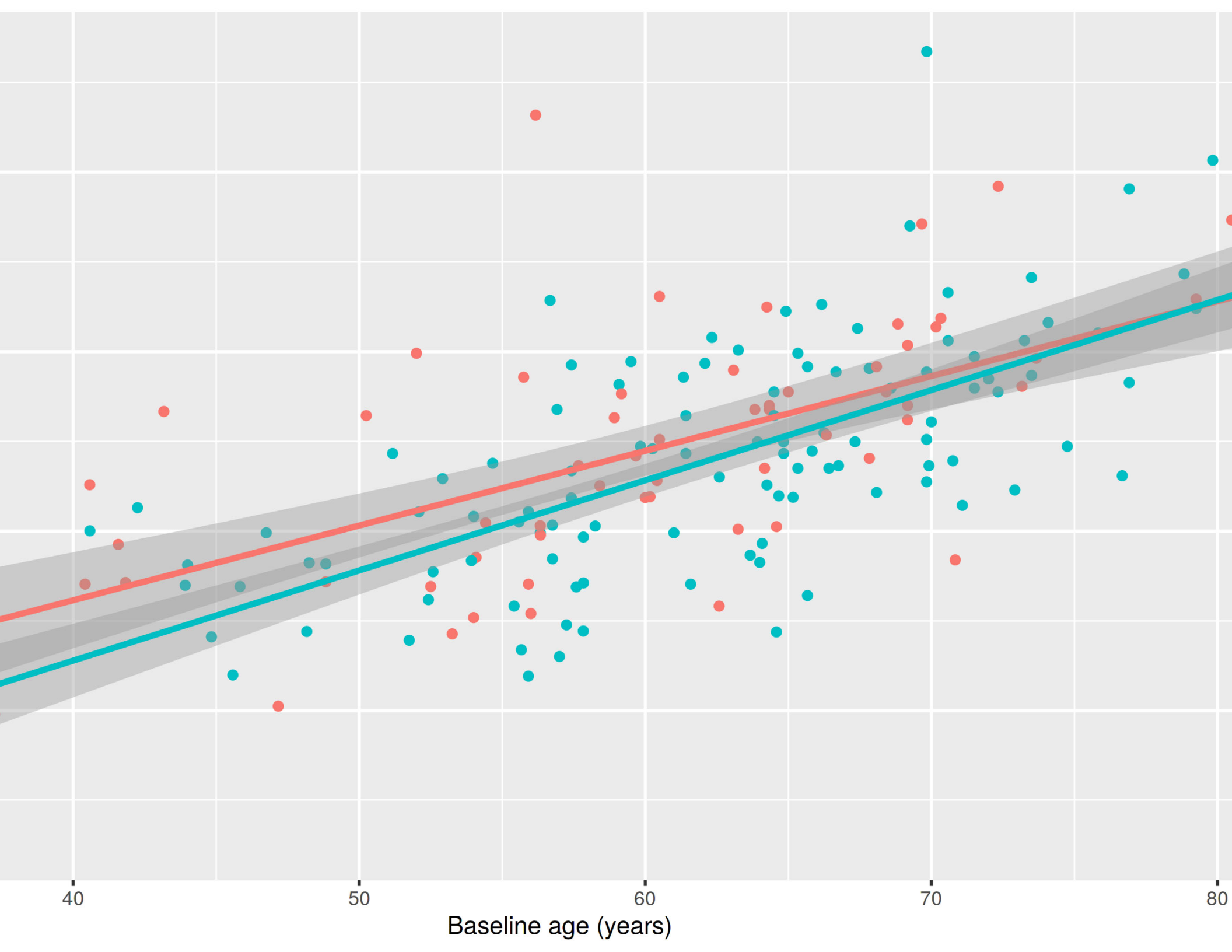

Supplement: Photo [file NIHMS1682170-supplement-Photo.pdf]
